# Probiotic *Lactobacillus* sp. inhibit growth, biofilm formation and gene expression of caries‐inducing *Streptococcus mutans*

**DOI:** 10.1111/jcmm.13496

**Published:** 2018-01-08

**Authors:** Reham Wasfi, Ola A. Abd El‐Rahman, Mai M. Zafer, Hossam M. Ashour

**Affiliations:** ^1^ Department of Microbiology and Immunology Faculty of Pharmacy October University for Modern Sciences and Arts (MSA) Giza Egypt; ^2^ Department of Microbiology and Immunology Faculty of Pharmacy Al‐Azhar University (Girls) Cairo Egypt; ^3^ Department of Microbiology and Immunology Faculty of Pharmacy Ahram Canadian University (ACU) Giza Egypt; ^4^ Department of Biological Sciences College of Arts and Sciences University of South Florida St. Petersburg St. Petersburg FL USA; ^5^ Department of Microbiology and Immunology Faculty of Pharmacy Cairo University Cairo Egypt

**Keywords:** probiotic Lactobacillus, *Streptococcus mutans*, biofilm, dental caries

## Abstract

*Streptococcus mutans* contributes significantly to dental caries, which arises from homoeostasic imbalance between host and microbiota. We hypothesized that *Lactobacillus* sp. inhibits growth, biofilm formation and gene expression of *Streptococcus mutans*. Antibacterial (agar diffusion method) and antibiofilm (crystal violet assay) characteristics of probiotic *Lactobacillus* sp. against *Streptococcus mutans* (ATCC 25175) were evaluated. We investigated whether *Lactobacillus casei* (ATCC 393), *Lactobacillus reuteri* (ATCC 23272), *Lactobacillus plantarum* (ATCC 14917) or *Lactobacillus salivarius* (ATCC 11741) inhibit expression of *Streptococcus mutans* genes involved in biofilm formation, quorum sensing or stress survival using quantitative real‐time polymerase chain reaction (qPCR). Growth changes (OD600) in the presence of pH‐neutralized, catalase‐treated or trypsin‐treated *Lactobacillus* sp. supernatants were assessed to identify roles of organic acids, peroxides and bacteriocin. Susceptibility testing indicated antibacterial (pH‐dependent) and antibiofilm activities of *Lactobacillus* sp. against *Streptococcus mutans*. Scanning electron microscopy revealed reduction in microcolony formation and exopolysaccharide structural changes. Of the oral normal flora, *L. salivarius* exhibited the highest antibiofilm and peroxide‐dependent antimicrobial activities. All biofilm‐forming cells treated with *Lactobacillus* sp. supernatants showed reduced expression of genes involved in exopolysaccharide production, acid tolerance and quorum sensing. Thus, *Lactobacillus* sp. can inhibit tooth decay by limiting growth and virulence properties of *Streptococcus mutans*.

## Introduction

Dental caries is a common chronic oral disease that can affect the health of adults and children 1. A number of studies demonstrated correlations between poor oral health and heart diseases 2, 3. Dental caries is an endogenous disease that results from homoeostatic imbalance between the host and microbiota 4. The shift of non‐pathogenic micro‐organism from commensalism to parasitism resulted from changes in the oral environment due to poor hygiene, smoking, systemic diseases and decrease in saliva flow 1, 5. *Streptococcus mutans* has been identified as a main contributor to dental caries 6.

The oral cariogenic biofilm formation occurs through phases that start by early colonization of pellicle by non‐mutans *Streptococci*. This phase creates a favourable area for the growth of *Streptococcus mutans* and initial biofilm formation 7. *Streptococcus mutans* possesses virulence factors that contribute to caries formation such as:

- Production of acid that damages dental hard tissues 8;
- An agmatine deiminase system and F‐ATPase encoded by the *aguBDAC* operon 9 and *atpD* gene 10, which are major components in acid‐adaptive response that contribute to the aciduric characteristics.
- The ability to synthesize exopolysaccharides (EPS) from sucrose by the action of multiple glucosyltransferases (Gtfs) encoded by the genes *gtfb*,* gtfc*,* gtfd*, in addition to fructosyltransferase encoded by *sacB (ftf*) gene. The glucosyltransferase and fructosyltransferase enzymes catalyse the synthesis of extracellular glucan and fructan polymers from sucrose, respectively 11. The EPS formed is thought to play dual roles in promoting microbial adherence to surface in addition to protecting embedded bacteria 12. These virulence factors work under the control of quorum‐sensing systems. The two‐component signal transduction systems (TCSTS), including *comCDE* and *vicRKX*, are among the regulatory networks that regulate gene expression in response to stimuli from the surrounding environment and are thus essential for bacterial survival and virulence modulation 13, 14.

Caries management strategies include the use of conventional physical removal of plaque and the reduction of bacterial population by chlorhexidine. Other interventions include maintaining the oral ecosystem by probiotics 15. Probiotic bacteria are live micro‐organisms that can confer health benefits to the host when administered in sufficient amounts 16. Most probiotics are Gram‐positive bacteria that belong to the genera *Lactobacillus* or *Bifidobacterium* 17.

Lactobacilli (LB) constitute part of the oral microbiota and can be linked to the oral health status of the individual 18. They comprise about 1% of the cultivable oral microbiota. The most common LB strains isolated from oral microbiota include *L. casei*,* L. paracasei*,* L. plantarum*,* L. rhamnosus*,* L. fermentum*,* L. acidophilus* and *L. salivarius* 19. Isolated LB strains from subjects without dental caries have a significantly increased capacity to inhibit the growth of *Streptococcus mutans* compared with the strains isolated from subjects with active caries. Thus, probiotic LB does have a therapeutic anticaries potential 20, 21, 22. Stamatova and Meurman 23.

There could be universal mechanisms by which probiotics impact oral pathogens. Generally, probiotics are believed to compete with pathogens for space and nutrients but have mostly unknown mechanisms of action. These may include impacts on the production of lactic acid, peroxide or bacteriocin in addition to possible immunomodulatory activities 24. We hypothesized that *Lactobacillus* sp. inhibits the growth, biofilm formation and gene expression of *Streptococcus mutans*. We then studied the mechanisms by which the probiotic *Lactobacillus* sp. antagonizes *Streptococcus mutans*.

## Materials and methods

### Bacterial strains, media and growth conditions

Four *Lactobacillus* sp. namely: *Lactobacillus casei* subspecies *casei* (ATCC 393), *Lactobacillus reuteri* (ATCC 23272), *Lactobacillus plantarum* subspecies *plantarum* (ATCC 14917) and *Lactobacillus salivarius* (ATCC 11741) were selected to study their effect on *Streptococcus mutans* (ATCC 25175) isolated from carious dentine. *Lactobacillus* sp. and *Streptococcus mutans* were cultured in deMan, Rogosa and Sharpe (MRS) and brain–heart infusion (BHI) media (Oxoid, Hampshire, Thermo Fisher Scientific, UK), respectively, at 37°C under anaerobic conditions using Oxoid Anaerogen^®^ sachets (Thermo Fisher Scientific, UK).

### Preparation of spent culture supernatant (SCS)

The spent culture supernatant (SCS) for each *Lactobacillus* sp. strain was prepared according to Lin *et al*. 25, and then the supernatant was filtered using 0.45‐μm filters (Millipore, Bedford, MA, USA). The supernatant was divided into four portions. One portion was left untreated, and the other three portions were treated to eliminate the effect of organic acids, hydrogen peroxide and bacteriocin. The effect of organic acid was neutralized by adjusting the pH of SCS to 6.5 with 1 N NaOH. The other two portions were treated with 1 mg/ml trypsin (Sigma‐Aldrich, USA) and 0.5 mg/ml catalase (Sigma‐Aldrich, C1345, USA) to eliminate the effect of bacteriocin and hydrogen peroxide, respectively 26. Treated and untreated supernatants were stored at −20°C.

### The agar diffusion method for antimicrobial screening of *Lactobacillus* sp

The antibacterial activity of *Lactobacillus* sp. on *Streptococcus mutans* was assessed using an agar diffusion method adapted from the one used by Cadirci and Citak 27. *Streptococcus mutans* was incubated in Brain–Heart Infusion (BHI) at 37°C for 24 hrs. Melted BHI agar medium held at 45°C was inoculated with *Streptococcus mutans* at a concentration equivalent to McFarland 0.5 standard (1.5 × 10^8^ CFU/ml). Wells of 7 mm diameter were filled by 100 μl of SCS. Inhibition zones were measured in millimetres after incubating the plates anaerobically at 37°C for 24 hrs. The same test was performed using *Lactobacillus* sp. whole bacterial culture (WBC) instead of SCS, with a turbidity equivalent to McFarland 0.5.

### Antibacterial testing of treated and untreated SCS

To determine the antibacterial activity of the SCS, *Streptococcus mutans* was grown overnight at 37°C in BHI broth. The *Streptococcus mutans* culture was diluted with BHI broth medium to a turbidity equivalent to McFarland 0.5 (1.5 × 10^8^ cells/ml). Then, 100 μl of the *Streptococcus mutans* suspension and 100 μl of untreated supernatants were added to the wells of 96‐well microtitre plate in eight replicates for each Lactobacillus SCS (Greiner Bio‐One, KremsmÜnster, Austria). The plates were then incubated anaerobically at 37°C for 24 hrs. In control wells, the SCS was replaced by sterile MRS broth. The OD600 nm was recorded after incubation using microplate reader (Stat Fax^®^2100) 28. The same steps were repeated with treated supernatants to determine the change in antimicrobial activity after removing the effect of acidic pH, peroxides and bacteriocin.

### The effect of *Lactobacillus* sp. SCS on *Streptococcus mutans* adherence

This test was performed in a similar manner as the antimicrobial test using BHI medium supplemented with 0.2% sucrose. After incubation, supernatants were removed, plates were stained, and reduction in biofilm formation was evaluated by crystal violet assay as previously described 29.

### The effect of *Lactobacillus* sp. SCS on *Streptococcus mutans* preformed biofilm

An overnight culture of *Streptococcus mutans* was diluted to McFarland 0.5 in BHI supplemented with 0.2% sucrose. This culture was distributed in the 96‐well microtitre plate by the volume of 100 μl and incubated at 37°C for 24 hrs. Culture supernatant was removed, and wells were washed with sterile saline. A volume of 100 μl of untreated supernatant was added in each well and incubated at 37°C for 24 hrs. Reduction in biofilm formation was determined as previously described 29.

### Scanning electron microscopy (SEM) observation of dual‐*Streptococcus mutans*–*Lactobacillus* sp. biofilm

*Streptococcus mutans* and *Lactobacillus* sp. were cocultured overnight at 37°C in BHI and MRS broth respectively followed by dilution to a concentration equivalent to McFarland 0.5. A clean sterile cover slide was added to the wells of the six‐well plate (Greiner Bio‐One, KremsmÜnster, Austria). In each well, 250 μl of the *Streptococcus mutans* suspension and 250 μl of one of the *Lactobacillus* sp. suspension were added to 1.5 ml of BHI broth (supplemented with 0.2% sucrose) and incubated anaerobically at 37°C for 24 hrs.

A monospecies culture of *Streptococcus mutans* biofilm was similarly prepared except that we replaced the *Lactobacillus* sp. culture with uncultured MRS medium. Cover slides were gently washed with phosphate‐buffered saline (PBS) once, fixed and prepared for SEM observation (JSM‐7600F, JEOL) according to a previously published protocol 30.

### Extraction of total bacterial RNA

We studied the effect of *Lactobacillus* sp. filtered supernatant on *Streptococcus mutans* in the planktonic form and the biofilm form. *Streptococcus mutans* was grown overnight at 37°C in BHI broth and was diluted to McFarland 0.5. A volume of 250 μl *Streptococcus mutans* suspension and 250 μl of the SCS were added to 1.5 ml of BHI broth and were incubated anaerobically at 37°C for 24 hrs. In control wells, the *Lactobacillus* sp. supernatant was replaced by MRS broth 25. After incubation, culture suspension was removed from wells for RNA extraction from planktonic bacteria. Cells adhering to the plate wells were washed twice by sterile saline and then dislodged and suspended in saline by scraping into a centrifuge tube. The total RNA was isolated from *Streptococcus mutans* planktonic and adherent cells using Direct‐Zol RNA MiniPrep kit (Zymo Research, CA, USA) according to the manufacturer's instructions. The remaining DNA in RNA samples was treated by RNase‐free DNase I (New England Biolab, MA, USA) to eliminate DNA contamination. Agarose gel electrophoresis of RNA samples verified its integrity. RNA concentration and purity were determined by the ND‐1000 spectrophotometer (NanoDrop Technology; Wilmington, DE, USA). Finally, the SensiFast™ cDNA synthesis kit (Bioline, MA, USA) was used to reverse transcribe 1 μg of total RNA sample into cDNA.

### Quantitative real‐time polymerase chain reaction (qRT‐PCR) and data analysis

Using qPCR, we examined the effect of *Lactobacillus* sp. spent supernatant on the expression levels of ten target genes [*gtfb*,* gtfc*,* gtfd*,* sacB*, comC, *comD*,* vick*,* vicR*,* aguD* and *atpD*] involved in glucan production, fructan production, quorum sensing and acid tolerance in *Streptococcus mutans*. The primers used for amplification of *comC*,* comD* and *sacB (ftf)* genes were designed using the complete genome sequence of *Streptococcus mutans* ATCC 25175 obtained from the NCBI database (GenBank accession no. PRJNA179256) and used as the base for primer design. Primers for the qPCR used in the current study (Table 1) were synthesized by Invitrogen (Massachusetts, USA). Quantitative real‐time reverse transcription polymerase chain reaction (qRT‐PCR) was performed by Applied Biosystems StepOne™ Instrument using SensiFast™ SYBR Hi‐Rox Master (Bioline, Massachusetts. USA). All reactions (20 μl) were performed using three technical replicates. Each reaction mixture contained 100 ng cDNA and 400 nM primers per reaction. The RT‐PCR cycling conditions were as follows: one cycle with 95°C for 2 min.; then 40 cycles of denaturation at 95°C for 5 sec., annealing at 52–62°C (depending on primers used) for 10 sec., and extension and fluorescent data collection at 72°C for 20 sec. A dissociation curve was generated at the end of each reaction. In all qPCR runs, negative controls without template were run in parallel. The *16s rRNA* gene (housekeeping gene) was selected as the internal control based on the results of BestKeeper^®^ software tool 31. The relative mRNA levels of genes of interest were determined and normalized to the expression of the housekeeping gene using the ∆∆CT value analysis 32. The qPCR data were expressed as the fold change in expression levels of genes in *Streptococcus mutans* ATCC 25175 cells exposed to SCS of the four tested *Lactobacillus* sp. as compared to their levels in the untreated cells (calibrators). The changes in gene expression were tested in the *Streptococcus mutans* cells in the planktonic form and the biofilm‐forming state.

**Table 1:** List of oligonucleotide sequences, their annealing temperature and amplicon size

Target gene^a^	Oligonucleotide sequence 5′–3′	Ta (°C)	Amplicon size (bp)	References
*gtfb*	For. ACGAACTTTGCCGTTATTGTCA Rev. AGCAATGCAGCCAATCTACAA	52	96	74
*gtfc*	For. CTCAACCAACCGCCACTGTT Rev. GGTTTAACGTCAAAATTAGCTGTATTAG	52	136	74
*gtfd*	For. TGTCTTGGTGGCCAGATAAAC Rev. GAACGGTTTGTGCAGCAAGG	62	132	74
*sacB (ftf)*	For. CCTGCGACTTCATTACGATTGGTC Rev. ATTGGCGAACGGCGACTTACTC	62	103	This study
*comC*	For. TATCATTGGCGGAAGCGGAA Rev. TCCCCAAAGCTTGTGTAAAACT	56	74	This study
*comD*	For. CGCGATTGGAGCCTTTAG Rev. CCTGAAATTCAGTTAGCCTTT	52	133	This study
*vicK*	For. CACTTTACGCATTCGTTTTGCC Rev. CGTTCTTCTTTTTCCTGTTCGGTC	56	102	65
*vicR*	For. CGCAGTGGCTGAGGAAAATG Rev. ACCTGTGTGTGTCGCTAAGTGATG	56	157	65
*aguD*	For. ATCCCGTGAGTGATAGTATTTG Rev. CAAGCCACCAACAAGTAAGG	56	80	63
*atpD*	For. CGTGCTCTCTCGCCTGAAATAG Rev. ACTCACGATAACGCTGCAAGAC	62	85	63
16s rRNA^b^	For. CCTACGGGAGGCAGCAGTAG Rev. CAACAGAGCTTTACGATCCGAAA	52	101	75

Ta: annealing temperature.

a *gtfb*, encoding glucosyltransferase I; *gtfC*, glucosyltransferase SI; *gtfD*, glucosyltransferase S; *sacB(ftf)*, encoding levansucrase enzyme (fructosyltransferase); *comC*, competence stimulating peptide; *comD*, Putative histidine kinase of the competence regulon; *vicK*, Putative histidine kinase CovS VicK‐like protein; *vicR*, Putative response regulator CovR VicR‐like protein; *aguD*, Agmatine: putrescine antiporter; *atpD*, F‐ATPase beta‐subunit; *16s rRNA*, 16s ribosomal RNA gene sequence.

b 16s gene was used as an internal control.

### Immunomodulatory effect of probiotic *Lactobacillus* sp

Human peripheral blood mononuclear cells (hPBMCs) from healthy volunteers were treated with SCS of *Lactobacillus* sp. as previously described by Wu *et al*. 30. The concentrations of IFN‐γ and IL‐10 were determined using enzyme‐linked immunosorbent assay (ELISA) according to the manufacturer's instructions (CUSABIO, BIOTECH CO, USA). A written consent was obtained from each subject. The protocol was approved by the Ethics Committee of the Faculty of Pharmacy, October University for Modern Sciences and Arts.

### Statistics

Experimental results were analysed for statistical significance using GraphPad Prism (GraphPad, San Diego, CA, USA). A one‐way analysis of variance (ANOVA) was performed. Data comparisons were performed using either Dunnett's multiple comparison test or Tukey's multiple comparison test.

## Results

### Agar diffusion assay

The zone of inhibition produced by whole bacterial culture (WBC) (concentration 1.5 × 10^8^ cells/ml) was larger than that produced by spent culture supernatant (SCS) produced by equivalent concentration of cells. This indicates the higher antimicrobial effect of WBC as compared to the cell‐free filtered supernatant. According to the zone of inhibition diameter, the highest antimicrobial activities of *Lactobacillus* sp. were observed with *L. casei* and *L. reuteri*, whereas the lowest antimicrobial activities were observed with *L. plantarum* and *L. salivarius* (Table 2).

**Table 2:** Antimicrobial effect of *Lactobacillus* sp. whole bacterial culture and filtered supernatant on the growth of *Streptococcus mutans*

Strain	Zone of inhibition^a^ (mm)	
	Whole bacterial culture (WBC)^b^	Spent culture supernatant (SCS)^b^
*Lactobacillus casei*	23 ± 1	18 ± 1
*Lactobacillus reuteri*	23 ± 3	18 ± 2
*Lactobacillus plantarum*	19 ± 1	14 ± 1
*Lactobacillus salivarius*	19 ± 2	14 ± 1

a The values are arithmetic means ± S.D. of inhibition zones (mm).

b All results were significantly different from control (*P *<* *0.01).

### Antimicrobial effect of treated and untreated *Lactobacillus* sp. supernatant against *Streptococcus mutans*

**Figure 1:**
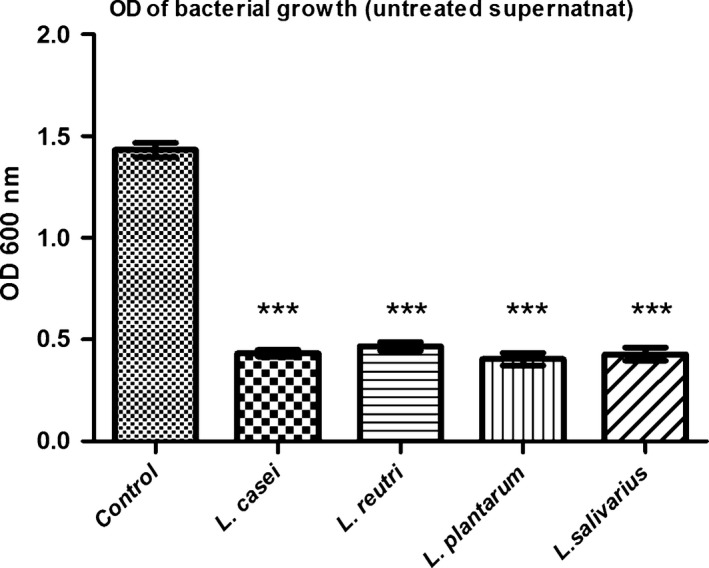
*Streptococcus mutans* growth in the presence of untreated *Lactobacillus* sp. supernatant. Optical density (OD) of *Streptococcus mutans* growth in the presence of untreated *Lactobacillus* sp. supernatants (*L. casei*,* L. reuteri*,* L. plantarum* and *L. salivarius*). Control: *Streptococcus mutans* growth in BHI broth. Untreated: spent culture supernatant (SCS). Data are expressed as the mean ± S.D., ***P <* *0.01 compared with *Streptococcus mutans* growth in BHI broth as control (Dunnett's multiple comparison test).

**Figure 2:**
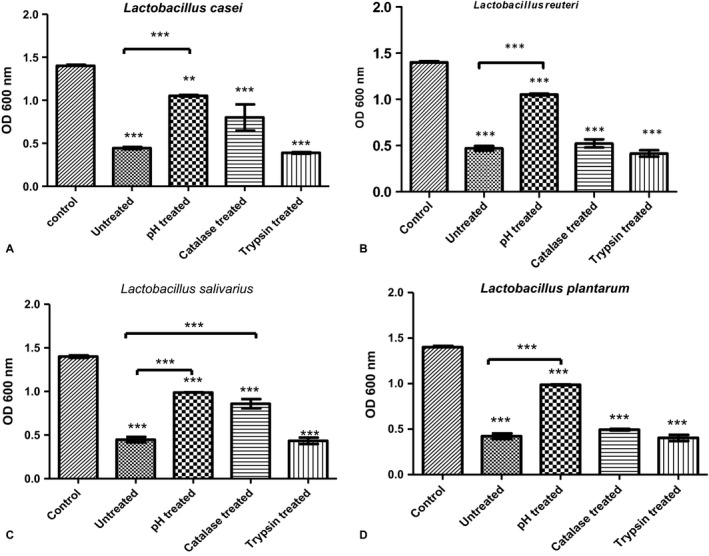
*Streptococcus mutans* growth in the presence of treated and untreated *Lactobacillus* sp. supernatant. Optical density (OD) of *Streptococcus mutans* growth in the presence of treated and untreated *Lactobacillus* sp. supernatants (*L. casei*,* L. reuteri*,* L. plantarum* and *L. salivarius*). Control: *Streptococcus mutans* grown in BHI broth. Untreated: Spent Culture Supernatant (SCS) of each strain supernatant, pH treated: supernatant with adjusted pH 6.5, catalase treated: supernatant after addition of 0.5 mg/ml catalase enzyme and trypsin treated: supernatant after addition of 1 mg/ml trypsin enzyme. Data are expressed as the mean ± S.D., **P <* *0.05 and ***P <* *0.01 compared with *Streptococcus mutans* grown in BHI broth as control (Tukey's multiple comparison test).

The untreated supernatants of the four *Lactobacillus* sp. showed strong significant inhibitory effect (Fig. 1) on the growth of *Streptococcus mutans* (*P *<* *0.01). There was no significant difference in the potency of the inhibitory effect between the four samples (*P *>* *0.05). After neutralizing the supernatant acidity, the antimicrobial effect was significantly reduced (*P *<* *0.01) compared with untreated supernatant, yet still showing significant reduction (*P *<* *0.05) in *Streptococcus mutans* growth (Fig. 2A, B, C and D). *Lactobacillus salivarius* was the only tested strain that showed significant reduction (*P *<* *0.05) in its antimicrobial effect on *Streptococcus mutans* after addition of catalase (Fig. 2D) indicating that peroxides contribute in its antimicrobial effect against *Streptococcus mutans*.

### Effect of *Lactobacillus* sp. filtered supernatants on *Streptococcus mutans* adherence and preformed biofilm

**Figure 3:**
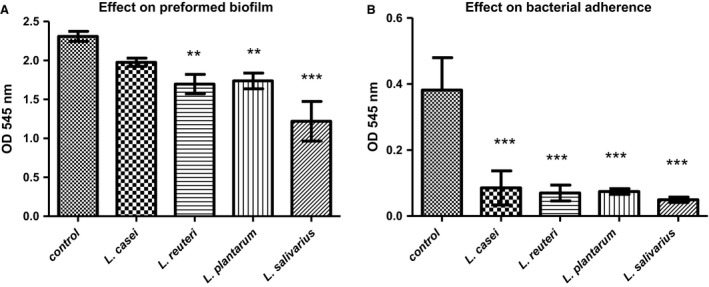
Effect of untreated supernatant on *Streptococcus mutans* (**A**) Effect of untreated supernatant on *Streptococcus mutans* adherence. (**B**) Effect of untreated supernatant on *Streptococcus mutans* preformed biofilm Optical density (OD 545 nm) of *Streptococcus mutans* biofilm in the presence of untreated *Lactobacillus* sp. supernatants (*L. casei*,* L. reuteri*,* L. plantarum* and *L. salivarius*). Control: *Streptococcus mutans* grown in BHI broth. Data are expressed as the mean ± S.D. **P <* *0.05, ***P <* *0.01 compared with control (Dunnett's multiple comparison test).

*Lactobacillus salivarius* supernatant caused significant reduction (*P *<* *0.01) in *Streptococcus mutans* adherence and preformed biofilm. Reduction percentages were 87% and 47%, respectively. The effect of *L. casei* supernatant was the least among tested supernatants on adherence as it showed no significant effect on the preformed biofilm. The *L. plantarum* and *L. reuteri* supernatant caused reduction in adherence with percentages of 81.7–80.5% and reduction in preformed biofilm with percentage of 26.5–24.7% (Fig. 3).

### Scanning electron microscope

**Figure 4:**
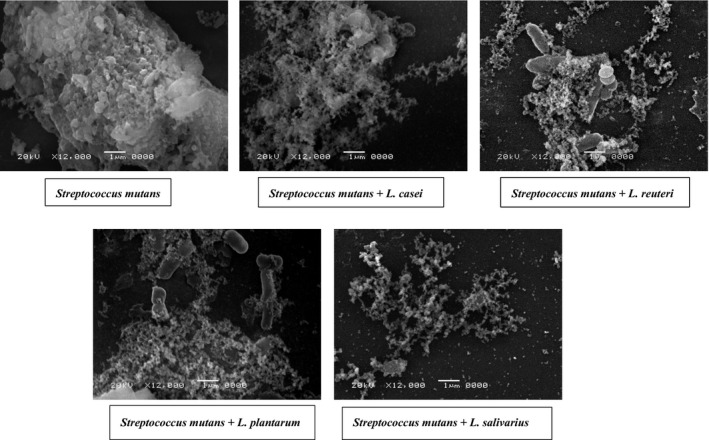
Scanning electron microscopy (SEM) of the biofilms. *Streptococcus mutans* was cocultured with *Lactobacillus* sp. as compared to *Streptococcus mutans* monoculture. The resulting biofilms were observed by SEM at 12,000× magnification.

As shown in Figure 4, the *Streptococcus mutans* appeared to form a compact, island‐like biofilm covered by large amounts of slime or network‐like structures. Changes in exopolysaccharides (EPS) matrix structure and quantity were observed in biofilm formed by coculture of *Streptococcus mutans* and different *Lactobacillus* sp. strains. Moreover, we observed fewer bacteria and smaller microcolonies attached to the surface.

### Analysis of qPCR results

We used qPCR to evaluate and compare the impact on *Streptococcus mutans* ATCC 25175 cells after exposure to four *Lactobacillus* sp. SCS (diluted 1:8 in BHI) overnight. The levels of expression of ten genes, that have been previously shown to be involved in virulence of the *S. mutans* in the planktonic and biofilm‐forming cells, were compared to the control untreated cells prepared under the same conditions without tested SCS. The selected genes included four genes involved in the two‐component signal transduction systems (TCSTS) [*comC*,* comD*,* vicK*,* vicR*], four genes involved in EPS formation [three of which are involved in glucan formation (*gtfB*,* gtfC* and *gtfD*), one gene is involved in fructan formation (*sacB* (*ftf)*)], and two genes associated with stress survival (*aguD*, and *atpD*).

**Figure 5:**
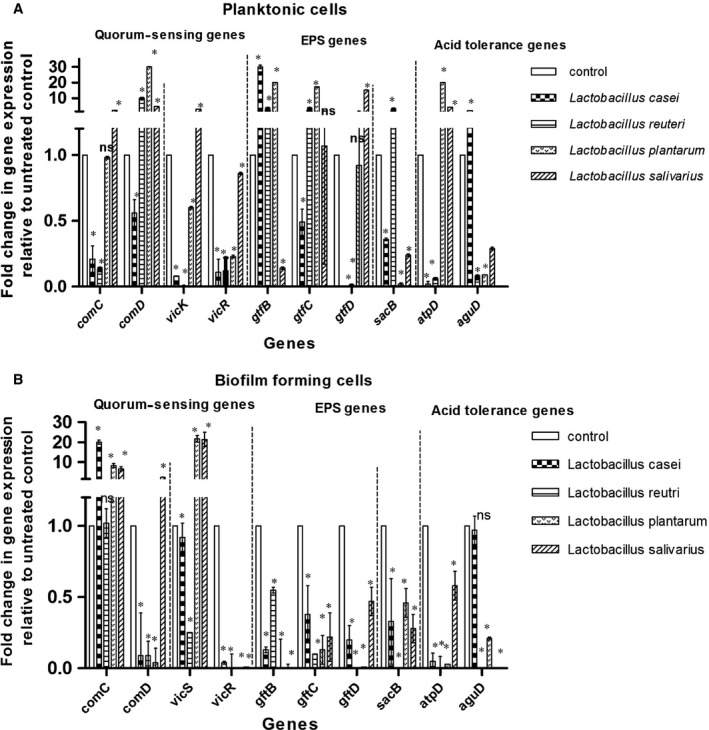
Alterations in gene expression profiles associated with exposure of *Streptococcus mutans* (ATCC 25175), in (**A**) planktonic form and (**B**) biofilm‐forming state, to the tested Spent culture supernatant (SCS) of *Lactobacillus casei* (ATCC 393), *Lactobacillus reuteri* (ATCC 23272), *Lactobacillus plantarum* (ATCC 14917) and *Lactobacillus salivarius* (ATCC 11741) as determined by qPCR. In each panel, fold change refers to the mean levels of gene expression across replicates, calculated using the ΔΔCt method relative to untreated control. Fold change = 2^−ΔΔCt^. Fold change (>1) indicates up‐regulation, (<1) indicates down‐regulation and fold change (~1) means insignificant change. Asterisks indicate statistically significant differences in the expression of each gene between treated samples and control, as analysed using the one‐way ANOVA with Dunnett's post‐testing for multiple testing (*P ≤* *0.01; *ns*, no significant difference). Error bars indicate standard deviation

As revealed by the one‐way ANOVA, there was an overall significant reduction (*P *<* *0.01) in the expression of most of the tested genes among the different groups, in both planktonic forms and biofilm‐forming cells. Dunnett's multiple comparison test was used to assess the significance of the difference between gene expression levels in target genes of exposed and control groups. As shown in Figure 5, few genes showed no significant difference (*P *>* *0.01) in expression as compared to the control under certain conditions. These genes are *comC* and *gtfD* in planktonic cells exposed to *L. plantarum* SCS, *gtfC* gene in planktonic cells exposed to *L. salivarius* SCS, and *comC* gene in the biofilm‐forming cells exposed to *L. reuteri* SCS.

The effect of SCS of different *Lactobacillus* sp. was variable on all tested TCSTS system genes. *L. salivarius* supernatant caused up‐regulation of the *vicK* gene by threefold to 21‐fold, in the planktonic and adherent cell forms, respectively. In planktonic cells, the expression of the *comC* gene, coding for competence‐stimulating peptide, was up‐regulated in the presence of *L. salivarius* supernatant only. Up‐regulation of the same gene was observed in biofilm‐forming cells treated with *L. casei*,* L. plantarum* and *L. salivarius*. The *comD* gene, coding for cognate histidine kinase receptor, was up‐regulated in the planktonic cells exposed to SCS of tested *Lactobacillus* sp. except for *L. casei*. On the other hand, it was down‐regulated in biofilm‐forming cells except those exposed to *L. salivarius* supernatant.

Significant reduction in gene expression of glucan (*gtfB*,* gtfC*,* gtfD*) and fructan (*sacB*) forming genes was observed in the adherent *Streptococcus mutans* cells in the presence of all tested SCS. The effects of the same supernatants were variable on the planktonic cells, as they showed significant up‐regulation (*P *<* *0.01) in *gtfB* and *gtfC* genes in the following cases: high up‐regulation in gene expression levels in presence of the supernatants of *L. casei* (30‐fold change in *gtfB* gene expression), and *L. plantarum* (20‐fold and 17‐fold change in *gtfB* and *gtfC* gene expression, respectively); moderate up‐regulation in gene expression of *gtfB* and *gtfC* genes in the presence of *L. reuteri* (2.5‐fold) supernatant. Significant up‐regulation (*P *<* *0.01) of the *gtfD* gene was observed in the presence of *L. salivarius* supernatant (15‐fold). Similarly, significant up‐regulation of the *sacB* (*ftf*) gene (2.5‐fold) was observed in the presence of *L. reuteri* supernatant.

Stress response genes (*atpD* and *aguD*) were down‐regulated in biofilm‐forming cells in the presence of all tested SCS. In planktonic forms, these two genes showed significant reduction (*P *<* *0.01) in expression except in two cases: The first is the *atpD* gene in the presence of *L. plantarum* and *L. salivarius* supernatants, and second is the *aguD* gene in the presence of *Lactobacillus casei*.

### Immunomodulatory activities of *Lactobacillus* sp

The SCS of *Lactobacillus* sp. was incubated with hPBMCs isolated from healthy volunteers for 48 hrs. The production levels of the immunostimulatory IFN‐γ and immunoregulatory IL‐10 cytokines were measured by ELISA. All *Lactobacillus* sp. standard strains stimulated hPBMCs to produce IFN‐γ higher than untreated controls. In contrast, IL‐10 concentrations were reduced after treating hPBMC with *Lactobacillus* sp. supernatants (Table 3).

**Table 3:** Effect of filtered Lactobacillus supernatant on interferon‐γ (IFN‐γ) production and interleukin‐10 (IL‐10) production in human peripheral blood mononuclear cells (hPBMCs) using enzyme‐linked immunosorbent assay (ELISA)

Sample	Cytokine concentration (pg/ml)^a^	
	IFN‐γ Mean ± S.D.	IL‐10 Mean ± S.D.
Control	15 ± 1	78 ± 1
*L. casei*	23.1 ± 0.5	63.5 ± 0.4
*L. reuteri*	31.2 ± 0.3	51.4 ± 0.5
*L. plantarum*	54.2 ± 0.5	27.3 ± 0.64
*L. salivarius*	49.3 ± 0.4	38.7 ± 0.6

a All results showed significant difference from control (*P *<* *0.01).

## Discussion

Dental caries is one of the most common diseases worldwide. The oral microbiota is composed of over 700 bacterial taxa 33. Under certain conditions, bacteria like *Streptococcus mutans* can be pathogenic and cause dental caries*. Streptococcus mutans* is a major contributor to dental caries development due to its virulence factors including the ability to synthesize extracellular polysaccharide and the ability to produce acidic metabolites 8.

*Lactobacillus* sp. constitute a main constituent of the microbiota in our oral cavity 34. *Lactobacillus* sp. probiotics have been proven to be efficient in treating certain gastrointestinal disorders 35. *Lactobacillus* sp. probiotics could possibly control dental caries using similar mechanisms that can play against *Streptococcus mutans* invasion strategies 8. This is because *Lactobacillus* sp. were shown to be able to produce organic acids, hydrogen peroxide, bacteriocins and adhesion inhibitors 36.

The *Lactobacillus* sp. used in this study were *L. casei* subspecies *casei* (ATCC 393), *L. reuteri* (ATCC 23272), *L. plantarum* subsp. *Plantarum* (ATCC 14917) and *L. salivarius* (ATCC 11741). These strains were chosen because they caused reduction in dental caries in previous studies including: *Lactobacillus casei* 37, 38, *Lactobacillus reuteri* 39, 40, 41, *Lactobacillus plantarum* 42 and *Lactobacillus salivarius* 43, 44. The precise mechanisms by which this happens are still unclear. Thus, the aim of the study was to assess mechanisms by which *Lactobacillus* sp. can control dental caries. We tested the effect of these four *Lactobacillus* sp. on the growth, adherence, biofilm formation and gene expression of *Streptococcus mutans* (ATCC 25175), in addition to the immunomodulatory effect.

The antimicrobial screening of the four tested strains of *Lactobacillus* using the agar diffusion method revealed differences in antimicrobial activity between different strains as determined by the size of the zone of inhibition. The highest effect was detected by *L. casei*, and *L. reuteri*, followed by *L. salivarius* and *L. plantarum*. Lactobacillus WBC caused higher antimicrobial effect on *Streptococcus mutans* than their corresponding SCS of the same *Lactobacillus* species. The difference in zone of inhibition caused by WBC compared to SCS may suggest that the presence of living metabolically active *Lactobacillus* sp. cells in WBC could result in the production of active antimicrobial agents in response to stimuli 45. The tested *Lactobacillus* sp. strains caused significant reduction in the microbial growth of *Streptococcus mutans* in BHI broth, as determined by the change in OD 600. At the same time, there was no significant difference between different *Lactobacillus* species regardless of their metabolic pattern. Strict homofermentative organisms such as *Lactobacillus salivarius*, facultative heterofermentative organisms such as *Lactobactobacillus casei* and *Lactobacillus plantarum*, and obligate heterofermentative organisms such as *Lactobacillus reuteri* showed similar antimicrobial effects.

To determine the effect of organic acids, hydrogen peroxide and bacteriocin produced by tested *Lactobacillus* sp., their effect was demolished by neutralization, catalase and trypsin addition, respectively. Neutralization of SCS to pH 6.5 significantly reduced the antimicrobial effect of the tested SCS. The low pH is an important factor for growth inhibition, and it is important for the production of bacteriocin 46. *Streptococcus mutans* is an acidogenic bacteria, that is produce organic acid as end product for sugar fermentation, and it is an aciduric bacteria, that is can tolerate acid in the plaque environment, hence, it can survive under acidic conditions 47. The acid tolerance genes, such as *atpD* and *aguD* genes, allow *Streptococcus mutans* to carry out metabolic processes at low‐pH values 48. The observed reduction in gene expression of *atpD*,* aguD*, in *Streptococcus mutans*, can decrease its acid tolerance, which can lead to bacteriostasis and eventual death 49. Anticaries agents such as the natural compounds α‐mangostin and catechin epigallocatechin gallate can down‐regulate the *atpD* and *aguD* genes 10, 50.

It was observed that neutralized SCS caused lower reduction in microbial growth than untreated SCS, but yet neutralized supernatant still showed significant reduction in *Streptococcus mutans* growth when compared to control. This suggests the influence of other antimicrobial agents such as hydrogen peroxide, bacteriocin, 51 and biosurfactant 52 that contribute with acid to growth inhibition.

Some *Lactobacillus* sp. have the ability to produce hydrogen peroxide, which can be toxic to organisms lacking hydrogen peroxide‐scavenging enzymes such as *Streptococcus mutans* 53. Adding catalase to *Lactobacillus* sp. supernatant caused reduction in the antimicrobial effect against *Streptococcus mutans*, but the significant reduction was observed only with *Lactobacillus salivarius* (ATCC 11741). This indicates that hydrogen peroxide contribution in antimicrobial activity of the tested *Lactobacillus* sp. is low except for *L. salivarius* supernatant.

*Streptococcus mutans* has been shown to initiate a response to various adverse environmental stressors, including oxidative stress, and acidic pH, by actively producing competence‐stimulating peptide (CSP) encoded by the *comC* gene 54. In our study, the expression of the *comC* was up‐regulated in biofilm‐forming cells compared with the untreated control. This was in contrast to *comD* which was down‐regulated in biofilm‐forming cells treated with *L. casei*,* L. reuteri* or *L. plantarum*. The antimicrobial testing of *L. salivarius* supernatant on *Streptococcus mutans* demonstrated the influence of supernatant pH and peroxide in the antimicrobial activity. Thus, the production of these stress factors by this strain might explain the significant up‐regulation in *comC* and *comD* genes in both the planktonic and biofilm‐forming *Streptococcus mutans* cells treated with this supernatant. Biofilm formed of *Streptococcus mutans* having single mutation in *comC*,* comD* and *comE*, or the triple mutation of *comCDE* showed different biofilm architecture in comparison with the wild‐type strain 55. This might explain the difference in biofilm formed by the coculture of *Streptococcus mutans* and *Lactobacillus* sp. as observed by SEM due to difference in *comCDE* expression.

*Lactobacillus* sp. can produce bacteriocin or bacteriocin‐like polypeptides that have a small molecular weight of <10 kD. In our study, trypsin‐treated supernatant showed no significant difference from the untreated SCS on *Streptococcus mutans* growth. This result indicates the low production of bacteriocin by *Lactobacillus* sp. This may not be the only explanation though because bacteriocin production by *Lactobacillus* sp. has been reported in several previous studies carried on *L. casei* 56, *L. reuteri* 57, *L. plantarum* 58 and *L. salivarius* 59.

*Streptococcus mutans* contributing to dental caries usually exists in the biofilm form inside the oral cavity. EPS of *Streptococcus mutans* contribute to dental caries by helping develop an oral biofilm in addition to forming a barrier against chemical agents. Therefore, therapeutic agents that target the biofilm can be the most suitable for dental caries prevention.

The cariogenic properties of *Streptococcus mutans* biofilms are regulated by various essential genes 60. Thus, the expression of representative biofilm‐associated genes was investigated. The genes studied included genes for sucrose‐dependent adhesion such as *gtfb*,* gtfC* 52 and *sacB (ftf*) 60. It also included two systems for controlling biofilms: (i) The *vicRKX* operon regulating the expression of virulence‐associated genes responsible for regulating the synthesis of polysaccharides, including *gtfBCD*,* sacB*, and polysaccharide‐binding sites as *gbpB* 61, (ii) *comCDE* quorum‐sensing system 48. In addition, it included genes for synthesis of insoluble glucan (*gtfB*,* gtfC*), soluble glucan (*gtfD*) 62 and fructan polymers (*sacB*) 61. Finally, it included genes responsible for acid tolerance (*aguD* and *atpD*) 63, 64.

The SCS of the four tested *Lactobacillus* sp. caused reduction in *Streptococcus mutans* biofilm with variable degrees. The highest reduction observed was with the supernatants of *L. salivarius* (87% for *Streptococcus mutans* adherence, and 47% for *Streptococcus mutans* preformed biofilm). The *vicR* gene was down‐regulated in planktonic forms and biofilm‐forming *Streptococcus mutans* exposed to all tested SCS. On the other hand, the *vicK* gene was down‐regulated upon the exposure of *Streptococcus mutans* to *L. casei* and *L. reuteri* supernatants. This down‐regulation might explain the reduction in *Streptococcus mutans* adherence and preformed biofilm as demonstrated by the SEM results. The *vicKRX* system has a significant influence on biofilm formation, and the null mutation in the *vicK* and *vicR* genes can cause aberrant biofilms which are easily removed 65. The *vicRKX* system regulates the glucosyltransferase‐encoding genes, and thus, mutation in this system can cause a significant decrease in *gtfD* gene expression, as well as increased expression of the *gtfB* gene 66. Changes in the *gtfB* and *gtfD* gene expressions, due to a mutated *vicRKX* system, were observed in planktonic *Streptococcus mutans* in the presence of *L. casei*,* L. reuteri* and *L. plantarum* supernatants, which caused a reduction in both *vicK* and *vicR* genes. In biofilm‐forming cells, both *gtfB* and *gtfD* genes were down‐regulated in the presence of all tested *Lactobacillus* sp. despite reduction in *vicK* and *vicR* genes. This could be attributed to the influence of factors other than *vicKRX* on the *gtf* genes such as: *luxS* (AI‐2 autoinducer‐coding synthesis), *ropA* (encoding for the trigger factor) and RegM (the catabolite‐repression regulator in *Streptococcus mutans*) 67, in addition to biosurfactants produced by *Lactobacillus* sp. which could reduce *gtfB*,* gtfC* and *gtf*D expressions in *Streptococcus mutans* 68.

*Gtf* genes that code for glucosyltransferase enzyme are primary virulence factors for *Streptococcus mutans* and thus can be a selective drug target for prevention of cariogenic biofilms. The *Lactobacillus* sp. supernatant‐induced altered gene expression indicates a promising anticaries effect. *In vitro* studies indicated that *gtfB* and *gtfC* were essential for the sucrose‐dependent attachment of *Streptococcus mutans* cells to hard surfaces and for microcolonies formation, but *gtfD* was not essential 52. In the biofilm‐forming bacteria, the expression of the three glucosyltransferase genes (*gtfB*,* gtfC* and *gtfD*) and the fructosyltransferase genes *sacB* (*ftf*) showed significant down‐regulation as compared to the control group of untreated biofilm‐forming cells. Glucosyltransferase S, encoded by g*tfB*, synthesizes insoluble glucan 67 and allows cell clustering 69. Thus, mutant *Streptococcus mutans* strains, defective in *gtfB*, are less cariogenic than their parent strains 70. The disruption of insoluble glucans synthesis can induce a reduction in biofilm formation, which can influence the pathogenesis 63. Thus, the *gtfB* expression reduction could explain the highest antibiofilm effect produced by *L. salivarius* on *Streptococcus mutans* adherence (87% reduction) and on preformed biofilm (47% reduction). The promising results of *L. salivarius* supernatant on *Streptococcus mutans* biofilms may indicate its possible anticaries effect. Thus, restoring the oral microenvironment with *L. salivarius* might be effective in preventing the colonization of periodontopathic bacteria 30. The difference in expression of *gtfB* and *gtfC* genes, in our study, indicates that there is no common promoter for them. Ullrich reported the potential presence of independent promoters for both genes 71. Low‐pH value increases the expression of the *gtfBC* gene but reduces *sacB* gene expression, which can lead to high‐biomass biofilms 48. The reduced *Streptococcus mutans* adherence and EPS formation in presence of SCS of *L. casei*,* L. reuteri* and *L. plantarum* despite the up‐regulation in the expression of the *gtfb* genes could be attributed to reduction in enzymatic function rather than reduction in gene expression 63.

The effect of *Lactobacillus* sp. on the production of IL‐10 and IFN‐γ was studied. IL‐10 is an immunosuppressive cytokine that is normally up‐regulated in inflamed pulp by bacterial infection to prevent the spread of inflammation 72. In our study, all tested *Lactobacillus* sp. supernatants inhibited IL‐10 production. To the best of our knowledge, this is the first study to show reduced IL‐10 production in response to *Lactobacillus* sp. This warrants further investigation. IFN‐γ is a pro‐inflammatory cytokine that synergizes with TNFα in increasing the microbicidal capacity of macrophages 73. The tested *Lactobacillus* sp. induced higher levels of IFN‐γ which can signify more robust innate and potentially adaptive immune responses at the site of infection.

The study showed that *Lactobacillus* sp. can inhibit tooth decay and control dental caries. This possible anticaries effect could be attributed to: (i) the inhibitory effect on *Streptococcus mutans* growth which were mainly due to organic acid generation and peroxide production; (ii) reduction in cell adherence and preformed biofilm; (iii) down‐regulation in several *Streptococcus mutans* virulence genes including acid tolerance genes (*atpD* and *aguD* genes), EPS‐producing genes (*gtfBCD* and *sacB*) and quorum‐sensing genes (*vicKR* and *comCD*); (iv) immunomodulatory effect due to the induction of IFN‐γ production and inhibition of IL‐10 production.

## Author contributions

Dr. Reham Wasfi, Dr. Ola A. Abd El‐Rahman, Dr. Mai M. Zafer and Dr. Hossam M. Ashour contributed to the design of the study, performance of experiments, analysis of the results and writing of the manuscript.

## Conflict of interest

The authors declare no competing financial interests.
